# Supplementary material for: Diarrhea in Yemeni children under five: A multi-level analysis of population-based surveys, 1991–2022
Source: PLoS Negl Trop Dis. 2025 Jul 30;19(7):e0013297. doi: 10.1371/journal.pntd.0013297 (PMC12310048; doi:10.1371/journal.pntd.0013297)
Supplement: S1 Table — (DOCX) [file pntd.0013297.s001.docx]

**S1 Table. Strengthening the reporting of observational studies in epidemiology (STROBE) checklist.**

|  | **Item No.** | **Recommendation** | **Main text page** |
| --- | --- | --- | --- |
| **Title and abstract** | 1 | (*a*) Indicate the study’s design with a commonly used term in the title or the abstract | Abstract |
|  |  | (*b*) Provide in the abstract an informative and balanced summary of what was done and what was found | Abstract |
| **Introduction** | | | |
| Background/rationale | 2 | Explain the scientific background and rationale for the investigation being reported | Introduction |
| Objectives | 3 | State specific objectives, including any prespecified hypotheses | Introduction |
| **Methods** | | | |
| Study design | 4 | Present key elements of study design early in the paper | Methods (‘Data source and study population’) |
| Setting | 5 | Describe the setting, locations, and relevant dates, including periods of recruitment, exposure, follow-up, and data collection | Methods (‘Data source and study population’) |
| Participants | 6 | (*a*) Give the eligibility criteria, and the sources and methods of selection of participants | Methods (‘Data source and study population’) |
| Variables | 7 | Clearly define all outcomes, exposures, predictors, potential confounders, and effect modifiers. Give diagnostic criteria, if applicable | Methods (‘Primary outcome’ & ‘Individual- and household-level factors’) |
| Data sources/ measurement | 8^*^ | For each variable of interest, give sources of data and details of methods of assessment (measurement). Describe comparability of assessment methods if there is more than one group | Methods (‘Primary outcome’ & ‘Individual- and household-level factors’) |
| Bias | 9 | Describe any efforts to address potential sources of bias | Methods, Statistical analysis (‘Estimation of diarrhea prevalence’) |
| Study size | 10 | Explain how the study size was arrived at | Methods (‘Data source and study population’) |
| Quantitative variables | 11 | Explain how quantitative variables were handled in the analyses. If applicable, describe which groupings were chosen and why | Methods (‘Individual- and household-level factors’ & ‘Meta-analysis of diarrhea prevalence in other countries with available DHS’ in Statistical analysis) & Tables 1-4 & Tables S2-S7 in Supplementary Appendix |
| Statistical methods | 12 | (*a*) Describe all statistical methods, including those used to control for confounding | Methods, Statistical analysis (‘Model selection and goodness of fit’, ‘Multilevel logistic regression analyses’ & ‘Meta-analysis of diarrhea prevalence in other countries with available DHS’) |
|  |  | (*b*) Describe any methods used to examine subgroups and interactions | Methods, Statistical analysis (‘Model selection and goodness of fit’) |
|  |  | (*c*) Explain how missing data were addressed | Methods, Statistical analysis (‘Missing data handling’) & Table 1 |
|  |  | (*d*) If applicable, describe analytical methods taking account of sampling strategy | Methods, Statistical analysis (‘Estimation of diarrhea prevalence’ & ‘Model selection and goodness of fit’) |
|  |  | (*e*) Describe any sensitivity analyses | Methods, Statistical analysis (‘Sensitivity analysis’) |
| **Results** | | | |
| Participants | 13^*^ | (a) Report numbers of individuals at each stage of study—eg numbers potentially eligible, examined for eligibility, confirmed eligible, included in the study, completing follow-up, and analysed | Results (‘Study population characteristics’) |
|  |  | (b) Give reasons for non-participation at each stage |  |
|  |  | (c) Consider use of a flow diagram |  |
| Descriptive data | 14^*^ | (a) Give characteristics of study participants (eg demographic, clinical, social) and information on exposures and potential confounders | Results (‘Study population characteristics’ & ‘Diarrhea prevalence’, paragraph 2) |
|  |  | (b) Indicate number of participants with missing data for each variable of interest |  |
| Outcome data | 15^*^ | Report numbers of outcome events or summary measures | Results (‘Diarrhea prevalence’, paragraph 1) & Table 1 & Figure 1 |
| Main results | 16 | (*a*) Give unadjusted estimates and, if applicable, confounder-adjusted estimates and their precision (eg, 95% confidence interval). Make clear which confounders were adjusted for and why they were included | Results (‘Associations with recent diarrhea’, ‘Neighborhood and household disparities’ & ‘Global diarrhea prevalence estimates’) & Tables 1-4 & Tables S2-S7 in Supplementary Appendix |
|  |  | (*b*) Report category boundaries when continuous variables were categorized | Tables 1-4 & Tables S2-S7 in Supplementary Appendix |
|  |  | (*c*) If relevant, consider translating estimates of relative risk into absolute risk for a meaningful time period | Not applicable |
| Other analyses | 17 | Report other analyses done—eg analyses of subgroups and interactions, and sensitivity analyses | Results (‘Sensitivity analysis’) & Tables S6 & S7 in Supplementary Appendix |
| **Discussion** | | | |
| Key results | 18 | Summarise key results with reference to study objectives | Discussion, paragraphs 1-12 |
| Limitations | 19 | Discuss limitations of the study, taking into account sources of potential bias or imprecision. Discuss both direction and magnitude of any potential bias | Discussion (‘Strengths and limitations’, paragraphs 2-4) |
| Interpretation | 20 | Give a cautious overall interpretation of results considering objectives, limitations, multiplicity of analyses, results from similar studies, and other relevant evidence | Discussion (‘Conclusions for action’) |
| Generalisability | 21 | Discuss the generalisability (external validity) of the study results | Discussion (‘Strengths and limitations’) |
| **Other information** | |  | |
| Funding | 22 | Give the source of funding and the role of the funders for the present study and, if applicable, for the original study on which the present article is based | Funding & Acknowledgements |

^*^Give information separately for cases and controls in case-control studies and, if applicable, for exposed and unexposed groups in cohort and cross-sectional studies.
