# Supplementary material for: Diarrhea in Yemeni children under five: A multi-level analysis of population-based surveys, 1991–2022
Source: PLoS Negl Trop Dis. 2025 Jul 30;19(7):e0013297. doi: 10.1371/journal.pntd.0013297 (PMC12310048; doi:10.1371/journal.pntd.0013297)
Supplement: S5 Table — (DOCX) [file pntd.0013297.s005.docx]

**S5 Table. Multilevel logistic regression analyses investigating associations with recent diarrhea in children under 5 years of age in Yemen 2022 MICS.**

| **Characteristics** | **Model 1 (Null Model)**^*^ | | **Model 2**^*^ | | **Model 3**^*^ | | **Model 4**^*^ | | **Model 5**^*^ | |
| --- | --- | --- | --- | --- | --- | --- | --- | --- | --- | --- |
|  | aOR^†^ (95% CI^†^) | p-value | aOR^†^ (95% CI^†^) | p-value | aOR^†^ (95% CI^†^) | p-value | aOR^†^ (95% CI^†^) | p-value | aOR^†^ (95% CI^†^) | p-value |
| **I. Individual-level factors** |  |  |  |  |  |  |  |  |  |  |
| Sex of child |  |  |  |  |  |  |  |  |  |  |
| Male | -- | -- | 1.00 |  | -- | -- | 1.00 |  | 1.00 |  |
| Female | -- | -- | 0.87 (0.80-0.95) | 0.001 | -- | -- | 0.87 (0.80-0.94) | 0.001 | 0.84 (0.75-0.95) | 0.006 |
| Current age of child (Months) |  |  |  |  |  |  |  |  |  |  |
| 0-11 | -- | -- | 1.00 |  | -- | -- | 1.00 |  | 1.00 |  |
| 12-23 | -- | -- | 1.46 (1.29-1.65) | <0.001 | -- | -- | 1.46 (1.29-1.65) | <0.001 | 1.73 (1.42-2.11) | <0.001 |
| 24-35 | -- | -- | 0.85 (0.75-0.96) | 0.007 | -- | -- | 0.84 (0.74-0.94) | 0.004 | 0.82 (0.68-0.98) | 0.033 |
| 36-47 | -- | -- | 0.68 (0.59-0.79) | <0.001 | -- | -- | 0.68 (0.59-0.78) | <0.001 | 0.55 (0.45-0.68) | <0.001 |
| 48-59 | -- | -- | 0.54 (0.47-0.61) | <0.001 | -- | -- | 0.54 (0.47-0.61) | <0.001 | 0.38 (0.31-0.46) | <0.001 |
| Mother's education |  |  |  |  |  |  |  |  |  |  |
| None | -- | -- | 1.00 |  | -- | -- | 1.00 |  | 1.00 |  |
| Basic | -- | -- | 1.02 (0.90-1.16) | 0.733 | -- | -- | 1.06 (0.94-1.20) | 0.334 | 1.19 (1.00-1.41) | 0.053 |
| Intermediate-Advanced | -- | -- | 0.92 (0.81-1.04) | 0.192 | -- | -- | 1.06 (0.93-1.20) | 0.375 | 1.21 (1.01-1.45) | 0.042 |
| **II. Household-level factors** |  |  |  |  |  |  |  |  |  |  |
| Place of residence |  |  |  |  |  |  |  |  |  |  |
| Urban | -- | -- | -- | -- | 1.00 |  | 1.00 |  | 1.00 |  |
| Rural | -- | -- | -- | -- | 0.80 (0.67-0.95) | 0.014 | 0.80 (0.67-0.96) | 0.015 | 0.69 (0.54-0.89) | 0.005 |
| Region |  |  |  |  |  |  |  |  |  |  |
| South Yemen | -- | -- | -- | -- | 1.00 |  | 1.00 |  | 1.00 |  |
| North Yemen | **--** | -- | -- | -- | 2.79 (2.38-3.27) | <0.001 | 2.84 (2.41-3.34) | <0.001 | 4.60 (3.61-5.86) | <0.001 |
| No. of individuals in household |  |  |  |  |  |  |  |  |  |  |
| 1-5 | -- | -- | -- | -- | 1.00 |  | 1.00 |  | 1.00 |  |
| 6-10 | -- | -- | -- | -- | 0.97 (0.86-1.08) | 0.539 | 1.00 (0.89-1.12) | 0.957 | 1.04 (0.89-1.20) | 0.646 |
| 11-15 | -- | -- | -- | -- | 0.80 (0.67-0.94) | 0.007 | 0.80 (0.67-0.95) | 0.010 | 0.77 (0.64-0.94) | 0.009 |
| >15 | -- | -- | -- | -- | 0.69 (0.57-0.84) | <0.001 | 0.70 (0.58-0.86) | 0.001 | 0.68 (0.52-0.88) | 0.004 |
| Wealth index |  |  |  |  |  |  |  |  |  |  |
| Lowest | -- | -- | -- | -- | 1.00 |  | 1.00 |  | 1.00 |  |
| Second | -- | -- | -- | -- | 1.09 (0.90-1.32) | 0.381 | 1.08 (0.89-1.31) | 0.430 | 0.91 (0.73-1.12) | 0.367 |
| Middle | -- | -- | -- | -- | 0.94 (0.75-1.18) | 0.618 | 0.93 (0.74-1.17) | 0.525 | 0.67 (0.51-0.87) | 0.003 |
| Fourth | -- | -- | -- | -- | 0.86 (0.66-1.12) | 0.274 | 0.84 (0.65-1.11) | 0.219 | 0.57 (0.41-0.79) | 0.001 |
| Highest | -- | -- | -- | -- | 0.59 (0.44-0.80) | 0.001 | 0.57 (0.42-0.77) | <0.001 | 0.31 (0.21-0.46) | <0.001 |
| Cooking place |  |  |  |  |  |  |  |  |  |  |
| House | -- | -- | -- | -- | 1.00 |  | 1.00 |  | 1.00 |  |
| Separate building | -- | -- | -- | -- | 1.06 (0.94-1.20) | 0.354 | 1.06 (0.94-1.20) | 0.321 | 1.07 (0.91-1.26) | 0.403 |
| Other | -- | -- | -- | -- | 0.70 (0.36-1.37) | 0.302 | 0.76 (0.40-1.44) | 0.399 | 0.93 (0.33-2.62) | 0.886 |
| ***WASH-related factors*** |  |  |  |  |  |  |  |  |  |  |
| Source of drinking water^‡^ |  |  |  |  |  |  |  |  |  |  |
| Unimproved | -- | -- | -- | -- | 1.00 |  | 1.00 |  | 1.00 |  |
| Improved | -- | -- | -- | -- | 1.06 (0.90-1.24) | 0.491 | 1.07 (0.91-1.25) | 0.433 | 1.11 (0.91-1.36) | 0.282 |
| Time to water source (Minutes) |  |  |  |  |  |  |  |  |  |  |
| On premises | -- | -- | -- | -- | 1.00 |  | 1.00 |  | 1.00 |  |
| ≤30 | -- | -- | -- | -- | 1.27 (1.08-1.50) | 0.003 | 1.28 (1.09-1.51) | 0.003 | 1.38 (1.12-1.70) | 0.002 |
| >30 | -- | -- | -- | -- | 1.20 (1.01-1.43) | 0.040 | 1.20 (1.01-1.44) | 0.041 | 1.30 (1.04-1.64) | 0.023 |
| Type of toilet facility^§^ |  |  |  |  |  |  |  |  |  |  |
| Unimproved | -- | -- | -- | -- | 1.00 |  | 1.00 |  | 1.00 |  |
| Improved | -- | -- | -- | -- | 0.84 (0.74-0.95) | 0.007 | 0.83 (0.73-0.95) | 0.006 | 0.79 (0.67-0.93) | 0.005 |
| Random effects estimates | | | | | | | | | | |
| PSU variance (95 CI %^†^) | 0.57 (0.48-0.67) | | 0.59 (0.50-0.70) | | 0.32 (0.27-0.40) | | 0.34 (0.28-0.41) | | 0.65 (0.51-0.84) | |
| Household variance (95 CI %^†^) | -- | | -- | | -- | | -- | | 3.64 (3.00-4.41) | |
| ICC^¦^ PSU (95 CI %^†^) | 0.15 (0.13-0.17) | | 0.15 (0.13-0.18) | | 0.09 (0.08-0.11) | | 0.09 (0.08-0.11) | | 0.09 (0.07-0.11) | |
| ICC^¦^ Household (95 CI %^†^) | -- | | -- | | -- | | -- | | 0.57 (0.52-0.61) | |
| MOR PSU (95 CI %^†^) | 2.05 (1.93-2.18) | | 2.08 (1.95-2.21) | | 1.72 (1.63-1.81) | | 1.74 (1.65-1.84) | | 2.16 (1.95-2.37) | |
| MOR Household (95 CI %^†^) | -- | | -- | | -- | | -- | | 6.17 (5.09-7.25) | |
| Model fit statistics | | | | | | | | | | |
| Log pseudolikelihood | -12206.8 | | -11980.3 | | -11973.4 | | -11743.5 | | -10717.0 | |
| AIC^¶^ | 24417.5 | | 23978.5 | | 23980.9 | | 23534.9 | | 21484.1 | |
| BIC^¶^ | 24433.3 | | 24049.2 | | 24114.4 | | 23723.5 | | 21680.6 | |

Abbreviations: aOR, adjusted odds ratio; AIC, Akaike information criterion; BIC, Bayesian information criterion; CI, confidence interval; ICC, intraclass correlation coefficient; MICS, multiple indicator cluster survey; MOR, median odds ratio; No., number; PSU, primary sampling unit; WASH, Water/Sanitation/Hygiene.

^*^The construction of the five models is delineated as follows: Model 1 (Null Model), serves as the baseline, includes only the intercept. Model 2 includes only individual-level factors. Model 3 includes only household-level factors. Model 4 both individual-level and household-level factors. The first four models are two-level logistic regression models that assess variability within PSUs, with PSUs included at the second level. Model 5 introduces a third hierarchical level, positioning household units at the second level and PSUs at the third level. The selection of covariates for these models is based on the results of univariable regression analyses presented in Table 3.

^†^Estimates were calculated applying MICS sampling weights to account for the complex survey design of the MICS study.

^‡^In classifying water sources, "unimproved" refers to sources such as regular wells, unprotected water surfaces, rivers, tanker trucks, and containerized water, while "improved" refers to government and local network supplies, tube and pumped wells, rainwater, and bottled water.

^§^In classifying toilet facilities, "unimproved" refers to pit latrines, flush toilets without sewer connections, open-drain toilets, street toilets, bucket toilets, shared facilities, and open defecation, while "improved" refers to flush toilets with piped sewer connections or septic tank systems.

^¦^Higher ICC values indicate a stronger clustering effect.

^¶^A lower criterion indicates a better model fit.
