# Supplementary material for: Diarrhea in Yemeni children under five: A multi-level analysis of population-based surveys, 1991–2022
Source: PLoS Negl Trop Dis. 2025 Jul 30;19(7):e0013297. doi: 10.1371/journal.pntd.0013297 (PMC12310048; doi:10.1371/journal.pntd.0013297)
Supplement: S7 Table — (DOCX) [file pntd.0013297.s007.docx]

**S7 Table. Sensitivity analyses. Regression models examining additional factors potentially associated with recent diarrhea (birthweight, breastfeeding, and health card possession) in children under 3 years of age in the 2013 Yemen DHS and 2022 MICS.**

| **Year** | **2013 DHS** | | | | | **2022 MICS** | | | | |
| --- | --- | --- | --- | --- | --- | --- | --- | --- | --- | --- |
| **Characteristics** | **Univariable regression analysis** | | | **Multilevel regression analysis^*^** | | **Univariable regression analysis** | | | **Multilevel regression analysis^*^** | |
|  | OR^†^ (95% CI^†^) | p-value | F test p-value^‡^ | aOR^†^ (95% CI^†^) | p-value^§^ | OR^†^ (95% CI^†^) | p-value | F test p-value^‡^ | aOR^†^ (95% CI^†^) | p-value^§^ |
| **I. Individual-level factors** |  |  |  |  |  |  |  |  |  |  |
| Sex of child |  |  |  |  |  |  |  |  |  |  |
| Male | 1.00 |  | 0.158 | 1.00 |  | 1.00 |  | 0.160 | 1.00 |  |
| Female | 0.92 (0.81-1.03) | 0.158 |  | 0.82 (0.64-1.03) | 0.093 | 0.92 (0.82-1.03) | 0.160 |  | 0.89 (0.67-1.17) | 0.398 |
| Current age of child (Months) |  |  |  |  |  |  |  |  |  |  |
| 0-11 | 1.00 |  | <0.001 | 1.00 |  | 1.00 |  | <0.001 | 1.00 |  |
| 12-23 | 1.53 (1.36-1.73) | <0.001 |  | 2.00 (1.56-2.56) | <0.001 | 1.38 (1.23-1.55) | <0.001 |  | 2.22 (1.65-2.98) | <0.001 |
| Mother's education |  |  |  |  |  |  |  |  |  |  |
| None | 1.00 |  | 0.298 | -- | -- | 1.00 |  | 0.758 | -- | -- |
| Basic | 0.84 (0.67-1.06) | 0.149 |  | -- | -- | 0.95 (0.81-1.11) | 0.512 |  | -- | -- |
| Intermediate-Advanced | 0.93 (0.80-1.08) | 0.324 |  | -- | -- | 0.95 (0.82-1.11) | 0.557 |  | -- | -- |
| Birthweight (Kilograms) |  |  |  |  |  |  |  |  |  |  |
| ≤2 | 1.00 |  | 0.085 | 1.00 |  | 1.00 |  | 0.003 | 1.00 |  |
| >2 | 0.54 (0.30-0.96) | 0.035 |  | 0.26 (0.09-0.72) | 0.010 | 0.68 (0.44-1.05) | 0.082 |  | 0.69 (0.28-1.67) | 0.406 |
| Unweighted | 0.65 (0.38-1.11) | 0.116 |  | 0.26 (0.10-0.68) | 0.006 | 1.07 (0.75-1.52) | 0.721 |  | 0.61 (0.28-1.31) | 0.202 |
| Breastfeed |  |  |  |  |  |  |  |  |  |  |
| Never breastfed | 1.00 |  | 0.155 | 1.00 |  | 1.00 |  | 0.932 | -- | -- |
| Ever breastfed | 0.71 (0.45-1.14) | 0.155 |  | 0.27 (0.10-0.76) | 0.013 | 1.01 (0.79-1.29) | 0.932 |  | -- | -- |
| Health card |  |  |  |  |  |  |  |  |  |  |
| No | 1.00 |  | 0.031 | 1.00 |  | 1.00 |  | <0.001 | 1.00 |  |
| Yes | 1.15 (1.01-1.31) | 0.031 |  | 1.75 (1.33-2.30) | <0.001 | 0.67 (0.59-0.76) | <0.001 |  | 0.49 (0.36-0.66) | <0.001 |
| **II. Household-level factors** |  |  |  |  |  |  |  |  |  |  |
| Place of residence |  |  |  |  |  |  |  |  |  |  |
| Urban | 1.00 |  | 0.024 | 1.00 |  | 1.00 |  | <0.001 | 1.00 |  |
| Rural | 1.25 (1.03-1.52) | 0.024 |  | 1.02 (0.65-1.60) | 0.935 | 1.35 (1.14-1.60) | <0.001 |  | 0.87 (0.54-1.41) | 0.577 |
| Region |  |  |  |  |  |  |  |  |  |  |
| South Yemen | 1.00 |  | <0.001 | 1.00 |  | 1.00 |  | <0.001 | 1.00 |  |
| North Yemen | 1.94 (1.65-2.29) | <0.001 |  | 3.37 (2.25-5.03) | <0.001 | 3.30 (2.79-3.91) | <0.001 |  | 8.24 (4.87-13.96) | <0.001 |
| No. of individuals in household |  |  |  |  |  |  |  |  |  |  |
| 1-5 | 1.00 |  | 0.133 | 1.00 |  | 1.00 |  | <0.001 | 1.00 |  |
| 6-10 | 0.84 (0.72-0.98) | 0.025 |  | 0.62 (0.46-0.83) | 0.002 | 0.99 (0.85-1.15) | 0.865 |  | 0.78 (0.57-1.09) | 0.145 |
| 11-15 | 0.83 (0.68-1.01) | 0.066 |  | 0.60 (0.41-0.89) | 0.010 | 0.82 (0.68-1.00) | 0.046 |  | 0.56 (0.38-0.83) | 0.004 |
| >15 | 0.85 (0.62-1.17) | 0.317 |  | 0.95 (0.56-1.60) | 0.838 | 0.58 (0.45-0.74) | <0.001 |  | 0.38 (0.22-0.66) | <0.001 |
| Wealth index |  |  |  |  |  |  |  |  |  |  |
| Lowest | 1.00 |  | 0.001 | 1.00 |  | 1.00 |  | <0.001 | 1.00 |  |
| Second | 0.98 (0.81-1.17) | 0.796 |  | 0.86 (0.59-1.26) | 0.440 | 0.95 (0.79-1.15) | 0.617 |  | 0.82 (0.54-1.23) | 0.335 |
| Middle | 0.95 (0.77-1.19) | 0.671 |  | 1.07 (0.70-1.62) | 0.766 | 0.76 (0.62-0.93) | 0.007 |  | 0.54 (0.32-0.89) | 0.015 |
| Fourth | 0.78 (0.61-0.98) | 0.037 |  | 0.84 (0.49-1.43) | 0.521 | 0.70 (0.57-0.87) | 0.001 |  | 0.52 (0.28-0.96) | 0.035 |
| Highest | 0.64 (0.51-0.80) | <0.001 |  | 0.52 (0.27-1.00) | 0.049 | 0.41 (0.33-0.51) | <0.001 |  | 0.25 (0.12-0.53) | <0.001 |
| Cooking place |  |  |  |  |  |  |  |  |  |  |
| House | 1.00 |  | 0.953 | -- | -- | 1.00 |  | <0.001 | 1.00 |  |
| Separate building | 1.06 (0.91-1.22) | 0.470 |  | -- | -- | 1.35 (1.18-1.55) | <0.001 |  | 1.15 (0.84-1.57) | 0.382 |
| Other | 1.06 (0.83-1.36) | 0.645 |  | -- | -- | 2.09 (0.88-4.92) | 0.093 |  | 7.38 (1.17-46.42) | 0.033 |
| ***WASH-related factors*** |  |  |  |  |  |  |  |  |  |  |
| Source of drinking water^¦^ |  |  |  |  |  |  |  |  |  |  |
| Unimproved | 1.00 |  | <0.001 | 1.00 |  | 1.00 |  | <0.001 | 1.00 |  |
| Improved | 0.74 (0.64-0.86) | <0.001 |  | 0.77 (0.57-1.04) | 0.085 | 0.74 (0.64-0.86) | <0.001 |  | 1.21 (0.83-1.77) | 0.320 |
| Time to water source (Minutes) |  |  |  |  |  |  |  |  |  |  |
| On premises | 1.00 |  | <0.001 | 1.00 |  | 1.00 |  | <0.001 | 1.00 |  |
| ≤30 | 1.46 (1.01-2.09) | 0.042 |  | 1.97 (1.03-3.78) | 0.040 | 1.67 (1.42-1.96) | <0.001 |  | 1.48 (1.02-2.17) | 0.041 |
| >30 | 1.51 (1.20-1.91) | <0.001 |  | 2.62 (1.67-4.11) | <0.001 | 1.48 (1.24-1.76) | <0.001 |  | 1.20 (0.78-1.86) | 0.414 |
| Type of toilet facility^¶^ |  |  |  |  |  |  |  |  |  |  |
| Unimproved | 1.00 |  | 0.004 | 1.00 |  | 1.00 |  | <0.001 | 1.00 |  |
| Improved | 0.82 (0.72-0.94) | 0.004 |  | 0.93 (0.69-1.25) | 0.619 | 0.68 (0.59-0.78) | <0.001 |  | 0.66 (0.48-0.92) | 0.013 |
| Water treatment |  |  |  |  |  |  |  |  |  |  |
| No | 1.00 |  | 0.294 | -- | -- | 1.00 |  | 0.134 | 1.00 |  |
| Yes | 1.12 (0.91-1.39) | 0.294 |  | -- | -- | 1.16 (0.95-1.42) | 0.134 |  | 1.31 (0.85-2.01) | 0.217 |
| Random effects estimates | | | | | | | | | | |
| PSU variance (95 CI %^†^) | NA | | | 0.75 (0.43-1.29) | | NA | | | 1.20 (0.76-1.90) | |
| Household variance (95 CI %^†^) | NA | | | 6.93 (5.06-9.49) | | NA | | | 9.65 (7.02-13.27) | |
| ICC^£^ PSU (95 CI %^†^) | NA | | | 0.07 (0.04-0.11) | | NA | | | 0.09 (0.05-0.12) | |
| ICC^£^ Household (95 CI %^†^) | NA | | | 0.70 (0.63-0.76) | | NA | | | 0.77 (0.71-0.82) | |
| MOR PSU (95 CI %^†^) | NA | | | 2.28 (1.76-2.79) | | NA | | | 2.85 (2.16-3.53) | |
| MOR Household (95 CI %^†^) | NA | | | 12.31 (7.45-17.17) | | NA | | | 19.36 (10.22-28.50) | |

Abbreviations: aOR, adjusted odds ratio; CI, confidence interval; DHS, demographic and health survey; ICC, intraclass correlation coefficient; MICS, multiple indicator cluster survey; MOR, median odds ratio; NA, not applicable; No., number; OR, odds ratio; PSU, primary sampling unit; WASH, Water/Sanitation/Hygiene.

^*^A three-level logistic regression model was used for each survey round, incorporating individual-level and household-level factors at the first level, household units at the second level, and PSUs at the third level.

^†^Estimates were calculated applying DHS or MICS sampling weights to account for the complex survey design of the DHS or MICS study.

^‡^Covariates with p-value ≤0.2 in the univariable analysis were included in the multilevel analysis.

^§^Covariates with p-value <0.05 in the multivariable analysis were considered as showing statistically significant evidence for an association with recent diarrhea.

^¦^In classifying water sources, "unimproved" refers to sources such as regular wells, unprotected water surfaces, rivers, tanker trucks, and containerized water, while "improved" refers to government and local network supplies, tube and pumped wells, rainwater, and bottled water.

^¶^In classifying toilet facilities, "unimproved" refers to pit latrines, flush toilets without sewer connections, open-drain toilets, street toilets, bucket toilets, shared facilities, and open defecation, while "improved" refers to flush toilets with piped sewer connections or septic tank systems.

^£^Higher ICC values indicate a stronger clustering effect.
