# Supplementary material for: Diarrhea in Yemeni children under five: A multi-level analysis of population-based surveys, 1991–2022
Source: PLoS Negl Trop Dis. 2025 Jul 30;19(7):e0013297. doi: 10.1371/journal.pntd.0013297 (PMC12310048; doi:10.1371/journal.pntd.0013297)
Supplement: Attachment — Submitted filename: Reviewer report - PNTD-D-24-01198.pdf [file pntd.0013297.s008.pdf]

Review report

Manuscript Title: Diarrhea in Yemeni Children Under Five: A Multi-Level Analysis of Population-Based Surveys, 1991-2022

Manuscript ID: PNTD-D-24-01198

This manuscript presents a well-structured and methodologically sound epidemiological study examining trends and determinants of childhood diarrhea in Yemen over a 31-year period. Drawing on four nationally representative surveys (DHS 1991 and 2013; MICS 2006 and 2022), the authors employ a robust three-level multilevel logistic regression model to account for the hierarchical nature of the data. The study identifies key risk factors at both individual and household levels and offers long-term trend analysis, which is particularly relevant given Yemen's ongoing conflict and humanitarian crisis and the inclusion of both national and global comparisons further strengthens the significance of the study.

Minor comments

1. The study focuses on a country with severe health system challenges, yet no Yemeni researchers appear to be included among the authors. The authors should clarify whether local scholars were engaged in the study design, interpretation, or dissemination. If efforts to include them were made but hindered by any contextual challenges, this should be acknowledged.

2. The study contributes substantially to the literature by using multi-round survey data over three decades and identifying household vs. neighborhood-level disparities. The authors could strengthen their case for originality by explicitly stating in the introduction how their findings extend or differ from previous research in other conflict-affected.

3. The use of a three-level random-effects logistic regression model is a suitable methodological choice for analysing nested survey data; however, the manuscript lacks clarity on whether essential survey design features-such as clustering and stratification from DHS and MICS-were incorporated beyond the application of sampling weights. Moreover, it is unclear how these sampling weights were applied within the multilevel modelling framework. Given the complexity of the DHS and MICS survey designs, the authors should explicitly describe how both the design features and weights were handled in the analysis.

4. Some key child health indicators (e.g., vaccination status, dietary diversity) were not included in the main models. The rationale for their exclusion should be discussed, and if data were unavailable across all survey waves, this should be explicitly noted as a study limitation.

5. The odds of diarrhea in North Yemen were over 11 times higher than in the South in 1991- a remarkably large effect. The authors should discuss whether this reflects real disparities, survey design limitations, or possible data anomalies.

6. The inclusion of pooled diarrhea prevalence from 82 countries offers valuable context. The rationale for using the Freeman-Tukey transformation should be clarified. The authors should also clarify how survey year was handled in countries with multiple survey rounds.
7. The Abstract should highlight the novelty of using a three-level model and analysing trends over a three-decade period.
8. Including "Multilevel modelling" among the Keywords is recommended to improve the article’s discoverability.

Overall assessment

This is a well-structured manuscript that addresses an urgent public health issue in a fragile setting. I encourage the authors to address the points outlined above to strengthen the overall quality of the work.
