# Supplementary material for: Diarrhea in Yemeni children under five: A multi-level analysis of population-based surveys, 1991–2022
Source: PLoS Negl Trop Dis. 2025 Jul 30;19(7):e0013297. doi: 10.1371/journal.pntd.0013297 (PMC12310048; doi:10.1371/journal.pntd.0013297)
Supplement: Attachment — Submitted filename: Response to Reviewers.doc [file pntd.0013297.s010.doc]

**Diarrhea in Yemeni Children Under Five: A Multi-Level Analysis of Population-Based Surveys, 1991-2022**

REPLY TO EDITORIAL AND REVIEWERS' COMMENTS

We are grateful to the editor and reviewers for assessing our work and for their insightful and useful feedback and suggestions. Please find below a point-by-point reply addressing each of the comments. We have also incorporated these suggestions in the revised manuscript, as noted below. We would be pleased to address any additional matters, should that be necessary.

Note: All references to the revised manuscript pertain to the marked copies of the manuscript files including changes implemented through "track changes".

*Dear Dr. Tag,*

*Thank you for submitting your manuscript to PLOS Neglected Tropical Diseases. After careful consideration, we feel that it has merit but does not fully meet PLOS Neglected Tropical Diseases's publication criteria as it currently stands. Therefore, we invite you to submit a revised version of the manuscript that addresses the points raised during the review process.*

Comment: We thank the editor and editorial team for the time and effort put into handling this submission, the positive assessment of our work, and the constructive feedback on our manuscript that enriched it and improved its readability. Please find below a point-by-point reply addressing each of the comments.

*Please submit your revised manuscript within 30 days Jul 27 2025 11:59PM. If you will need more time than this to complete your revisions, please reply to this message or contact the journal office at* [*plosntds@plos.org*](mailto:plosntds@plos.org)*. When you're ready to submit your revision, log on to* [*https://www.editorialmanager.com/pntd/*](https://www.editorialmanager.com/pntd/) *and select the 'Submissions Needing Revision' folder to locate your manuscript file.*

*Please include the following items when submitting your revised manuscript:*

** A rebuttal letter that responds to each point raised by the editor and reviewer(s). You should upload this letter as a separate file labeled 'Response to Reviewers'. This file does not need to include responses to any formatting updates and technical items listed in the 'Journal Requirements' section below.*

Answer: These instructions have been noted and addressed as indicated.

** A marked-up copy of your manuscript that highlights changes made to the original version. You should upload this as a separate file labeled 'Revised Manuscript with Track Changes'.*

Answer: These instructions have been noted and addressed as indicated.

** An unmarked version of your revised paper without tracked changes. You should upload this as a separate file labeled 'Manuscript'.*

Answer: These instructions have been noted and addressed as indicated.

*If you would like to make changes to your financial disclosure, competing interests statement, or data availability statement, please make these updates within the submission form at the time of resubmission. Guidelines for resubmitting your figure files are available below the reviewer comments at the end of this letter.*

Answer: These instructions have been noted and addressed as indicated.

**Journal Requirements:**

1) *Please upload all main figures as separate Figure files in .tif or .eps format. For more information about how to convert and format your figure files please see our guidelines:*
   [*https://journals.plos.org/plosntds/s/figures*](https://journals.plos.org/plosntds/s/figures)

Answer: These instructions have been noted and addressed as indicated.

2) *We have noticed that you have uploaded Supporting Information files, but you have not included a list of legends. Please add a full list of legends for your Supporting Information files after the references list.*

Answer: These instructions have been noted and addressed as indicated (Page 41).

3) *Please amend your detailed Financial Disclosure statement. This is published with the article. It must therefore be completed in full sentences and contain the exact wording you wish to be published. State the initials, alongside each funding source, of each author to receive each grant. For example: "This work was supported by the National Institutes of Health (####### to AM; ###### to CJ) and the National Science Foundation (###### to AM).".
   If you did not receive any funding for this study, please simply state: “The authors received no specific funding for this work.”*

Answer: These instructions have been noted and addressed as indicated (Funding).

**Reviewer comments:**

Reviewer's Responses to Questions

***Key Review Criteria Required for Acceptance?*** *As you describe the new analyses required for acceptance, please consider the following:*

***Methods*** *-Are the objectives of the study clearly articulated with a clear testable hypothesis stated?

-Is the study design appropriate to address the stated objectives?

-Is the population clearly described and appropriate for the hypothesis being tested?

-Is the sample size sufficient to ensure adequate power to address the hypothesis being tested?

-Were correct statistical analysis used to support conclusions?

-Are there concerns about ethical or regulatory requirements being met?*

Reviewer #1:

*Manuscript Title; Diarrhea in Yemeni Children Under Five: A Multi-Level Analysis of Population-Based Surveys, 1991-2022
Manuscript Number: PNTD-D-24-01198
Authors; Zahir M. Tag, , Laith J. Abu-Raddad, , and Hiam Chemaitelly,
Reviewer; Mohamed A Daw, MD, MPS, Pharm D, PhD, Mrcpath(1A), FTCDI, Faculty of Medicine, University of Tripoli –Libya*

Comment: We thank the reviewer for the time and effort put into this review, the assessment of our work, and the constructive feedback on our manuscript that enriched it and improved its readability. Please find below a point-by-point reply addressing each of the reviewer's comments.

*Parachute research;
Though I have gone through the whole MS, I did no see any Yamani institute or even participant was involved in this study. This raise a major question regarding the gaining and reliability of the data involved in the study, which raise the issue of Parachute research.*

Answer: We apologize for the confusion regarding this point. The first author is Yemeni and conducted this study as part of a one-year internship at Weill Cornell Medicine–Qatar and as his Master of Science project, motivated by a strong personal and professional commitment to improving public health outcomes in Yemen. His national and cultural background informed the study's design, interpretation, and motivation. All survey data analyzed were collected by Yemeni institutions, including the Central Statistical Organization and the Ministry of Public Health and Population, with technical and financial support from the DHS Program, which operates under the USAID, and from UNICEF. We made repeated efforts to engage local institutions for additional data; however, due to access limitations and a lack of response—possibly related to the ongoing conflict—our requests went unanswered.

These clarifications have now been added to the Methods section (Methods, Page 5, Paragraph 5 and Page 6, Paragraph 1 and Paragraph 2) and Acknowledgments (Acknowledgements).

*Recommendation
Reject ; or resubmit based on;
The authors should include a person or a Yamani institute who should be involved in the study*

Answer: As indicated above, the first author is Yemeni and conducted this study as part of a one-year internship at Weill Cornell Medicine–Qatar and as his Master of Science project, motivated by a strong personal and professional commitment to improving public health outcomes in Yemen. His national and cultural background informed the study's design, interpretation, and motivation. All survey data analyzed were collected by Yemeni institutions, including the Central Statistical Organization and the Ministry of Public Health and Population, with technical and financial support from the DHS Program, which operates under the USAID, and from UNICEF. We made repeated efforts to engage local institutions for additional data; however, due to access limitations and a lack of response—possibly related to the ongoing conflict—our requests went unanswered.

These clarifications have now been added to the Methods section (Methods, Page 5, Paragraph 5 and Page 6, Paragraph 1 and Paragraph 2) and Acknowledgments (Acknowledgements).

Reviewer #2:

The second reviewer's comments were submitted as a separate attachment. For completeness, these comments have been copied below and addressed point by point.

*Review report*

*Manuscript Title: Diarrhea in Yemeni Children Under Five: A Multi-Level Analysis of Population-Based Surveys, 1991-2022*

*Manuscript ID: PNTD-D-24-01198*

*This manuscript presents a well-structured and methodologically sound epidemiological study examining trends and determinants of childhood diarrhea in Yemen over a 31-year period. Drawing on four nationally representative surveys (DHS 1991 and 2013; MICS 2006 and 2022), the authors employ a robust three-level multilevel logistic regression model to account for the hierarchical nature of the data. The study identifies key risk factors at both individual and household levels and offers long-term trend analysis, which is particularly relevant given Yemen’s ongoing conflict and humanitarian crisis and the inclusion of both national and global comparisons further strengthens the significance of the study.*

Comment: We thank the reviewer for the time and effort put into this review, the assessment of our work, and the constructive feedback on our manuscript that enriched it and improved its readability. Please find below a point-by-point reply addressing each of the reviewer's comments.

***Minor comments***

1. *The study focuses on a country with severe health system challenges, yet no Yemeni researchers appear to be included among the authors. The authors should clarify whether local scholars were engaged in the study design, interpretation, or dissemination. If efforts to include them were made but hindered by any contextual challenges, this should be acknowledged.*

Answer: We apologize for the confusion regarding this point. The first author is Yemeni and conducted this study as part of a one-year internship at Weill Cornell Medicine–Qatar and as his Master of Science project, motivated by a strong personal and professional commitment to improving public health outcomes in Yemen. His national and cultural background informed the study's design, interpretation, and motivation. All survey data analyzed were collected by Yemeni institutions, including the Central Statistical Organization and the Ministry of Public Health and Population, with technical and financial support from the DHS Program, which operates under the USAID, and from UNICEF. We made repeated efforts to engage local institutions for additional data; however, due to access limitations and a lack of response—possibly related to the ongoing conflict—our requests went unanswered.

These clarifications have now been added to the Methods section (Methods, Page 5, Paragraph 5 and Page 6, Paragraph 1 and Paragraph 2) and Acknowledgments (Acknowledgements).

2. *The study contributes substantially to the literature by using multi-round survey data over three decades and identifying household vs. neighborhood-level disparities. The authors could strengthen their case for originality by explicitly stating in the introduction how their findings extend or differ from previous research in other conflict-affected.*

Answer: Thank you for the valuable feedback and useful suggestion. We have added a sentence explicitly highlighting how our findings extend or differ from previous research conducted in other conflict-affected settings (Introduction, Page 5, Paragraph 3).

3. *The use of a three-level random-effects logistic regression model is a suitable methodological choice for analysing nested survey data; however, the manuscript lacks clarity on whether essential survey design features-such as clustering and stratification from DHS and MICS-were incorporated beyond the application of sampling weights. Moreover, it is unclear how these sampling weights were applied within the multilevel modelling framework. Given the complexity of the DHS and MICS survey designs, the authors should explicitly describe how both the design features and weights were handled in the analysis.*

Answer: We apologize for the lack of clarity regarding this point. We used a three-level random-effects logistic regression model, specifying random effects at the household and PSU levels to capture the hierarchical data structure. Individual-level sampling weights from DHS and MICS were applied within the model at the first level. This combination effectively accounts for clustering and the complex survey design, consistent with methodological standards from previous studies. These details have now been clarified in the Methods section (Methods, Page 8, Paragraph 4 and Page 9, Paragraph 1 and Paragraph 2).

4. *Some key child health indicators (e.g., vaccination status, dietary diversity) were not included in the main models. The rationale for their exclusion should be discussed, and if data were unavailable across all survey waves, this should be explicitly noted as a study limitation.*

Answer: Excellent point—thank you. Other child health indicators, including vaccination status and dietary diversity, were excluded from the main models because they were either not collected or exhibited substantial missing data across multiple survey waves. This point has now been clarified in the Discussion section (Discussion, Page 20, Paragraph 3).

5. *The odds of diarrhea in North Yemen were over 11 times higher than in the South in 1991- a remarkably large effect. The authors should discuss whether this reflects real disparities, survey design limitations, or possible data anomalies.*

Answer: Thank you for useful suggestion. This point has now been explained in the revised manuscript (Discussion, Page 18, Paragraph 3).

6. *The inclusion of pooled diarrhea prevalence from 82 countries offers valuable context. The rationale for using the Freeman-Tukey transformation should be clarified. The authors should also clarify how survey year was handled in countries with multiple survey rounds.*

Answer: Both of these points have now been clarified in the Methods section (Methods, Page 11, Paragraph 1 and Paragraph 2).

7. *The Abstract should highlight the novelty of* *using a three-level model and analysing trends over a three-decade period.*

Answer: This addition has now been incorporated into the Abstract (Abstract).

8. *Including "Multilevel modelling" among the Keywords is recommended to improve the article’s discoverability.*

Answer: Excellent point—thank you. This addition has now been incorporated into the Keywords (Keywords).

***Overall assessment***

*This is a well-structured manuscript that addresses an urgent public health issue in a fragile setting. I encourage the authors to address the points outlined above to strengthen the overall quality of the work.*

Answer: Thank you for the valuable feedback and useful suggestions. All indicated points have now been addressed as recommended.

Reviewer #3:

Comment: We thank the reviewer for the time and effort put into this review, the assessment of our work, and the constructive feedback on our manuscript that enriched it and improved its readability. Please find below a point-by-point reply addressing each of the reviewer's comments.

*1-Yes the study’s objectives are clearly stated. The authors aim to assess the prevalence, trends, and determinants of diarrhea among children under five in Yemen across a 30 year span. While the manuscript doesn’t formally state a hypothesis in the traditional “if-then”format, the analytical approach is driven by clear research questions focusing on associations between individual/household factors and diarrhea risk, making the investigation testable and hypothesis oriented in practice*

Answer: Thank you—we sincerely appreciate the reviewer's thoughtful and positive assessment of our work.

*2-Is the study design appropriate to address the stated objectives?
Absolutely. The use of nationally representative, cross sectional datasets (DHS and MICS) is well suited for identifying temporal trends and population level associations. The three level random effects and logistic regression is appropriate given the nested nature of the data (individuals within households within neighborhoods). Including both household and neighborhood clustering effects strengthens the credibility of the findings.*

Answer: We sincerely appreciate the reviewer's thoughtful and positive assessment of our work.

*3-Yes, the study population is well defined. The authors used data from four large national surveys, covering over 45,000 children under five across multiple decades. The sampling methodology (two-stage cluster design) is robust and clearly explained, ensuring representativeness across different regions and socioeconomic backgrounds.*

Answer: We sincerely appreciate the reviewer's thoughtful and positive assessment of our work.

*4-The sample sizes for each survey year are more than adequate to detect meaningful associations, with the 2022 MICS including nearly 20,000 children. This large sample ensures sufficient statistical power, especially given the multilevel structure of the analysis*

Answer: We sincerely appreciate the reviewer's thoughtful and positive assessment of our work.

*5-The authors applied a thoughtful and thorough statistical approach. The three level multivariable logistic regression model is well justified and executed, accounting for clustering at the household and PSU levels. Model fit comparisons (via AIC/BIC), checks for multicollinearity (VIF), and the use of sensitivity analyses all reflect sound statistical practice. The supplementary global metaanalysis comparing Yemen to other regions further contextualizes the findings*

Answer: We sincerely appreciate the reviewer's thoughtful and positive assessment of our work.

*6-There is no major concerns. The authors correctly note that ethical approval was not required due to the use of publicly available, de identified DHS and MICS datasets. They also appropriately acknowledge the sources of funding and confirm the absence of competing interests, which adds transparency*

Answer: We sincerely appreciate the reviewer's thoughtful and positive assessment of our work.

Reviewer #4:

Comment: We thank the reviewer for the time and effort put into this review, the assessment of our work, and the constructive feedback on our manuscript that enriched it and improved its readability. Please find below a point-by-point reply addressing each of the reviewer's comments.

*The study would benefit from incorporating real-time surveillance data from WHO’s Electronic Disease Early Warning System (eDEWS), which provides weekly updates on disease trends, including diarrhea outbreaks. Using current surveillance data would help validate the long-term trends identified and reflect the current situation more accurately.*

Answer: Thank you for this excellent suggestion. We attempted to do so; however, we were unable to access the primary eDEWS platform, as account creation is restricted, limiting our ability to obtain data for analysis. Nevertheless, we reviewed the available literature on the system's performance during the conflict in Yemen. While eDEWS has been valuable for outbreak detection, its application for longitudinal analysis is limited by several challenges, including delays in case verification, increasing dissemination lags, reliance on manual data entry, and incomplete data coverage . This point has now been discussed in the revised manuscript (Discussion, Page 20, Paragraph 4).

***Results*** *-Does the analysis presented match the analysis plan?

-Are the results clearly and completely presented?

-Are the figures (Tables, Images) of sufficient quality for clarity?*

Reviewer #1:

*Manuscript Title; Diarrhea in Yemeni Children Under Five: A Multi-Level Analysis of Population-Based Surveys, 1991-2022
Manuscript Number: PNTD-D-24-01198
Authors; Zahir M. Tag, , Laith J. Abu-Raddad, , and Hiam Chemaitelly,
Reviewer; Mohamed A Daw, MD, MPS, Pharm D, PhD, Mrcpath(1A), FTCDI, Faculty of Medicine, University of Tripoli –Libya*

Comment: We thank the reviewer for the time and effort put into this review, the assessment of our work, and the constructive feedback on our manuscript that enriched it and improved its readability. Please find below a point-by-point reply addressing each of the reviewer's comments.

*Parachute research;
Though I have gone through the whole MS, I did no see any Yamani institute or even participant was involved in this study. This raise a major question regarding the gaining and reliability of the data involved in the study, which raise the issue of Parachute research.*

Answer: We apologize for the confusion regarding this point. The first author is Yemeni and conducted this study as part of a one-year internship at Weill Cornell Medicine–Qatar and as his Master of Science project, motivated by a strong personal and professional commitment to improving public health outcomes in Yemen. His national and cultural background informed the study's design, interpretation, and motivation. All survey data analyzed were collected by Yemeni institutions, including the Central Statistical Organization and the Ministry of Public Health and Population, with technical and financial support from the DHS Program, which operates under the USAID, and from UNICEF. We made repeated efforts to engage local institutions for additional data; however, due to access limitations and a lack of response—possibly related to the ongoing conflict—our requests went unanswered.

These clarifications have now been added to the Methods section (Methods, Page 5, Paragraph 5 and Page 6, Paragraph 1 and Paragraph 2) and Acknowledgments (Acknowledgements).

*Recommendation
Reject ; or resubmit based on;
The authors should include a person or a Yamani institute who should be involved in the study*

Answer: As indicated above, the first author is Yemeni and conducted this study as part of a one-year internship at Weill Cornell Medicine–Qatar and as his Master of Science project, motivated by a strong personal and professional commitment to improving public health outcomes in Yemen. His national and cultural background informed the study's design, interpretation, and motivation. All survey data analyzed were collected by Yemeni institutions, including the Central Statistical Organization and the Ministry of Public Health and Population, with technical and financial support from the DHS Program, which operates under the USAID, and from UNICEF. We made repeated efforts to engage local institutions for additional data; however, due to access limitations and a lack of response—possibly related to the ongoing conflict—our requests went unanswered.

These clarifications have now been added to the Methods section (Methods, Page 5, Paragraph 5 and Page 6, Paragraph 1 and Paragraph 2) and Acknowledgments (Acknowledgements).

Reviewer #3:

Comment: We thank the reviewer for the time and effort put into this review, the assessment of our work, and the constructive feedback on our manuscript that enriched it and improved its readability. Please find below a point-by-point reply addressing each of the reviewer's comments.

*Yes, the analysis closely follows the plan outlined in the Methods section. The authors clearly indicate their intention to use multi-level logistic regression to examine individual and household level risk factors, accounting for clustering at the household and neighborhood (PSU) levels. This is consistently applied across all four survey rounds. The statistical techniques used including univariable screening, model selection via AIC/BIC, and the use of ICC and MOR to assess clustering are appropriate and align well with what was described up front. The inclusion of a sensitivity analysis and a global metaanalysis to provide context was also part of the methods and is well integrated into the final analysis.*

Answer: Thank you—we sincerely appreciate the reviewer's thoughtful and positive assessment of our work.

Reviewer #4:

Comment: We thank the reviewer for the time and effort put into this review, the assessment of our work, and the constructive feedback on our manuscript that enriched it and improved its readability. Please find below a point-by-point reply addressing each of the reviewer's comments.

*The results section discusses random-effects estimates (ICC & MOR) but lacks sufficient interpretation for non-statistical readers. Consider explaining ICC and MOR in more practical terms, particularly in relation to household vs. neighborhood disparities.*

Answer: Thank you for the useful suggestion. We have now added an interpretation of these measures, emphasizing how they reflect the relative contribution of household- and neighborhood-level factors to disparities in childhood diarrhea (Results, Page 15, Paragraph 1 and Paragraph 2).

***Conclusions*** *-Are the conclusions supported by the data presented?

-Are the limitations of analysis clearly described?

-Do the authors discuss how these data can be helpful to advance our understanding of the topic under study?

-Is public health relevance addressed?*

Reviewer #1:

*Manuscript Title; Diarrhea in Yemeni Children Under Five: A Multi-Level Analysis of Population-Based Surveys, 1991-2022
Manuscript Number: PNTD-D-24-01198
Authors; Zahir M. Tag, , Laith J. Abu-Raddad, , and Hiam Chemaitelly,
Reviewer; Mohamed A Daw, MD, MPS, Pharm D, PhD, Mrcpath(1A), FTCDI, Faculty of Medicine, University of Tripoli –Libya*

Comment: We thank the reviewer for the time and effort put into this review, the assessment of our work, and the constructive feedback on our manuscript that enriched it and improved its readability. Please find below a point-by-point reply addressing each of the reviewer's comments.

*Parachute research;
Though I have gone through the whole MS, I did no see any Yamani institute or even participant was involved in this study. This raise a major question regarding the gaining and reliability of the data involved in the study, which raise the issue of Parachute research.*

Answer: We apologize for the confusion regarding this point. The first author is Yemeni and conducted this study as part of a one-year internship at Weill Cornell Medicine–Qatar and as his Master of Science project, motivated by a strong personal and professional commitment to improving public health outcomes in Yemen. His national and cultural background informed the study's design, interpretation, and motivation. All survey data analyzed were collected by Yemeni institutions, including the Central Statistical Organization and the Ministry of Public Health and Population, with technical and financial support from the DHS Program, which operates under the USAID, and from UNICEF. We made repeated efforts to engage local institutions for additional data; however, due to access limitations and a lack of response—possibly related to the ongoing conflict—our requests went unanswered.

These clarifications have now been added to the Methods section (Methods, Page 5, Paragraph 5 and Page 6, Paragraph 1 and Paragraph 2) and Acknowledgments (Acknowledgements).

*Recommendation
Reject ; or resubmit based on;
The authors should include a person or a Yamani institute who should be involved in the study*

Answer: As indicated above, the first author is Yemeni and conducted this study as part of a one-year internship at Weill Cornell Medicine–Qatar and as his Master of Science project, motivated by a strong personal and professional commitment to improving public health outcomes in Yemen. His national and cultural background informed the study's design, interpretation, and motivation. All survey data analyzed were collected by Yemeni institutions, including the Central Statistical Organization and the Ministry of Public Health and Population, with technical and financial support from the DHS Program, which operates under the USAID, and from UNICEF. We made repeated efforts to engage local institutions for additional data; however, due to access limitations and a lack of response—possibly related to the ongoing conflict—our requests went unanswered.

These clarifications have now been added to the Methods section (Methods, Page 5, Paragraph 5 and Page 6, Paragraph 1 and Paragraph 2) and Acknowledgments (Acknowledgements).

Reviewer #3:

Comment: We thank the reviewer for the time and effort put into this review, the assessment of our work, and the constructive feedback on our manuscript that enriched it and improved its readability. Please find below a point-by-point reply addressing each of the reviewer's comments.

*Yes, the conclusions are well supported by the data. The findings from the multilevel regression models, trend analyses, and sensitivity checks all align with the central claims made by the authors particularly regarding the persistence and worsening burden of childhood diarrhea in Yemen, the importance of household level factors, and the growing disparities over time. The statistical associations are clearly presented, and the interpretation of adjusted odds ratios is appropriately cautious and grounded in the data.*

Answer: Thank you—we sincerely appreciate the reviewer's thoughtful and positive assessment of our work.

Reviewer #4:

Comment: We thank the reviewer for the time and effort put into this review, the assessment of our work, and the constructive feedback on our manuscript that enriched it and improved its readability. Please find below a point-by-point reply addressing each of the reviewer's comments.

*The study identifies risk factors but provides limited recommendations for policy action. Expand on potential WASH interventions: targeted sanitation programs, hygiene education, water infrastructure development, etc.*

Answer: Excellent suggestion—thank you. The conclusions have now been expanded to include policy recommendations, incorporating the suggested interventions as well as additional relevant strategies (Discussion, Page 21, Paragraph 1; Paragraph 2; and Paragraph 3).

*Discuss rural vs. urban intervention strategies to tailor solutions effectively.*

Answer: Thank you for the useful suggestion. This has now been incorporated to ensure the recommendations are both context-specific and actionable (Discussion, Page 21, Paragraph 2).

***Editorial and Data Presentation Modifications?*** *Use this section for editorial suggestions as well as relatively minor modifications of existing data that would enhance clarity. If the only modifications needed are minor and/or editorial, you may wish to recommend “Minor Revision” or “Accept”.*

Reviewer #1:

*Manuscript Title; Diarrhea in Yemeni Children Under Five: A Multi-Level Analysis of Population-Based Surveys, 1991-2022
Manuscript Number: PNTD-D-24-01198
Authors; Zahir M. Tag, , Laith J. Abu-Raddad, , and Hiam Chemaitelly,
Reviewer; Mohamed A Daw, MD, MPS, Pharm D, PhD, Mrcpath(1A), FTCDI, Faculty of Medicine, University of Tripoli –Libya*

Comment: We thank the reviewer for the time and effort put into this review, the assessment of our work, and the constructive feedback on our manuscript that enriched it and improved its readability. Please find below a point-by-point reply addressing each of the reviewer's comments.

*Parachute research;
Though I have gone through the whole MS, I did no see any Yamani institute or even participant was involved in this study. This raise a major question regarding the gaining and reliability of the data involved in the study, which raise the issue of Parachute research.*

Answer: We apologize for the confusion regarding this point. The first author is Yemeni and conducted this study as part of a one-year internship at Weill Cornell Medicine–Qatar and as his Master of Science project, motivated by a strong personal and professional commitment to improving public health outcomes in Yemen. His national and cultural background informed the study's design, interpretation, and motivation. All survey data analyzed were collected by Yemeni institutions, including the Central Statistical Organization and the Ministry of Public Health and Population, with technical and financial support from the DHS Program, which operates under the USAID, and from UNICEF. We made repeated efforts to engage local institutions for additional data; however, due to access limitations and a lack of response—possibly related to the ongoing conflict—our requests went unanswered.

These clarifications have now been added to the Methods section (Methods, Page 5, Paragraph 5 and Page 6, Paragraph 1 and Paragraph 2) and Acknowledgments (Acknowledgements).

*Recommendation
Reject ; or resubmit based on;
The authors should include a person or a Yamani institute who should be involved in the study*

Answer: As indicated above, the first author is Yemeni and conducted this study as part of a one-year internship at Weill Cornell Medicine–Qatar and as his Master of Science project, motivated by a strong personal and professional commitment to improving public health outcomes in Yemen. His national and cultural background informed the study's design, interpretation, and motivation. All survey data analyzed were collected by Yemeni institutions, including the Central Statistical Organization and the Ministry of Public Health and Population, with technical and financial support from the DHS Program, which operates under the USAID, and from UNICEF. We made repeated efforts to engage local institutions for additional data; however, due to access limitations and a lack of response—possibly related to the ongoing conflict—our requests went unanswered.

These clarifications have now been added to the Methods section (Methods, Page 5, Paragraph 5 and Page 6, Paragraph 1 and Paragraph 2) and Acknowledgments (Acknowledgements).

Reviewer #3:

*Overall this is a wellexecuted and highly relevant study with solid methodological rigor and clear public health implications. The manuscript is suitable for publication with only few minor revisions and editorial improvements to enhance clarity and readability*

Comment: We thank the reviewer for the time and effort put into this review, the assessment of our work, and the constructive feedback on our manuscript that enriched it and improved its readability. Please find below a point-by-point reply addressing each of the reviewer's comments.

*1-writing and language:
Some sections particularly in the Methods and Results are dense with technical detail. Consider simplifying or breaking up long paragraphs for better flow.*

Answer: Thank you for the helpful suggestion. We have now edited the Methods and Results sections by breaking down long paragraphs and refining the text where appropriate to improve readability and flow (Methods and Results).

*2- figures and tables:
Table 1 is comprehensive but quite lengthy. Consider splitting it into two parts (e.g., individual and household level variables) or moving some less critical rows to supplementary material to enhance focus*

Answer: We appreciate the reviewer's comment. During manuscript preparation, we carefully considered several formatting options, including splitting the table or relocating selected variables to the Supporting Information. However, after multiple iterations, we found that presenting all variables within a single table offered the most coherent and visually balanced layout. This approach also facilitates direct comparison of individual- and household-level characteristics, enhancing interpretability while minimizing the number of necessary tables—an important consideration given the multiple surveys analyzed in this study.

*3-terminology:
Ensure consistent terminology across the manuscript for WASH indicators (eg., unimproved toilet and unimproved toilet facilities) and geographic regions (eg., North Yemen and northern Yemen).*

Answer: Thank you for pointing this out. We have carefully reviewed the manuscript to ensure consistent use of terminology for WASH indicators and geographic regions, aligning terms with standard definitions and applying them uniformly throughout the text (Multiple instances throughout the manuscript).

Reviewer #4:

*Moderate Revision*

***Summary and General Comments*** *Use this section to provide overall comments, discuss strengths/weaknesses of the study, novelty, significance, general execution and scholarship. You may also include additional comments for the author, including concerns about dual publication, research ethics, or publication ethics. If requesting major revision, please articulate the new experiments that are needed.*

Reviewer #1: No comments

Reviewer #3:

*This manuscript gives a comprehensive and timely analysis of diarrhea prevalence among children under five in Yemen over a 30 year period, utilizing nationally representative DHS and MICS surveys. The authors apply multi level logistic regression models to explore both individual and household level factors that influencing diarrhea risk, while also comparing Yemen’s situation to global trends. The study is highly relevant given the ongoing humanitarian crisis in Yemen and offers important insights into public health disparities which is reminded me of what’s happening in sudan and south sudan because of the ongoing conflicts
2. Strengths
1-Robust dataset: The useing of four large scale, population based surveys across multiple decades provides solid ground for temporal analysis.
2-Methodological rigor: The application of three level random effects logistic regression is appropriate and adds depth, especially in capturing clustering effects.
3-Global context: The inclusion of global prevalence comparisons through DHS metaanalysis enhances the broader relevance of the findings.
4-Policy relevance: The findings are actionable and offer clear implications for improving WASH infrastructure and targeting vulnerable populations*

Comment: We thank the reviewer for the time and effort put into this review, the assessment of our work, and the constructive feedback on our manuscript that enriched it and improved its readability. Please find below a point-by-point reply addressing each of the reviewer's comments.

*A few minor weaknesses to consider:
1-The text, while thorough, can be dense in places. Streamlining certain sections and reducing redundancy would improve flow.*

Answer: Thank you for the helpful suggestion. To improve flow and readability, we have broken down dense paragraphs, simplified complex sentences to enhance clarity and reduce redundancy, and repositioned some content to ensure better logical progression (Multiple instances throughout the manuscript).

*2-While ethical approval is appropriately addressed, a brief note on how missing data were handled in practical terms (e.g., any imputation and listwise deletion) could add clarity.*

Answer: Thank you for the useful suggestion. This information has now been relocated and is now emphasized under a dedicated heading titled "Missing data handling" within the Statistical Analysis section to enhance clarity and overall organization (Methods, Page 10, Paragraph 3).

*In terms of novelety, this study fills a major gap in the literature by moving beyond small, hospital based studies to provide a national picture with longitudinal insights. The findings are significant for public health planning in Yemen and other conflict affected countries such as Sudan , South sudan , Syria and Palestine where access to water, sanitation, and healthcare is compromised.*

Answer: Thank you—we sincerely appreciate the reviewer's thoughtful and positive assessment of our work.

*There’s no concerns regarding publication ethics*

Answer: We sincerely appreciate the reviewer's thoughtful and positive assessment of our work.

Reviewer #4:

*This manuscript presents a well-conceived and methodologically rigorous study addressing the burden of childhood diarrhea in Yemen. The use of nationally representative data and advanced three-level random-effects logistic regression modeling strengthens the analysis. The findings are highly relevant for public health policy, particularly in settings affected by humanitarian crises. However, the manuscript would benefit from clarifications in statistical interpretation, stronger policy recommendations, and improved contextual analysis of ongoing health system interventions and external factors affecting disease prevalence.*

Comment: We thank the reviewer for the time and effort put into this review, the assessment of our work, and the constructive feedback on our manuscript that enriched it and improved its readability. Please find below a point-by-point reply addressing each of the reviewer's comments.

*The introduction contains excessive background detail—consider streamlining for better readability.*

Answer: Thank you for the helpful suggestion. We have now streamlined the Introduction by removing certain background details and condensing other text—particularly text related to Yemen—to enhance readability and maintain a clearer focus (Introduction, Page 4, Paragraph 4 and Page 5, Paragraph 1 and Paragraph 2).

*The study does not discuss vaccination programs, particularly rotavirus vaccine introduction in Yemen—a key preventive measure against diarrheal diseases. If available, information on coverage rates, effectiveness, and impact should be included to strengthen the disease prevention aspect of the analysis.*

Answer: Thank you for the excellent point. We have added a paragraph discussing the introduction of the rotavirus vaccine in Yemen, including available data on coverage rates (Discussion, Page 17, Paragraph 3).

*Yemen has undergone significant population displacement due to conflict, economic instability, and natural disasters. How has internal migration, refugee influx, or rural-to-urban shifts affected diarrhea prevalence and access to healthcare? Integrating mobility trends could provide a deeper understanding of geographic disparities in disease burden.*

Answer: Thank you for another excellent point. We have added a paragraph discussing the impact of internal displacement and rural-to-urban migration on diarrhea prevalence and access to healthcare in Yemen (Discussion, Page 18, Paragraph 4).

*PLOS authors have the option to publish the peer review history of their article (*[*what does this mean?*](https://track.editorialmanager.com/CL0/https:%2F%2Fjournals.plos.org%2Fplosntds%2Fs%2Feditorial-and-peer-review-process%23loc-peer-review-history/1/010f019716293912-3089958a-ff36-4e9f-b883-3a4a69a06bd7-000000/K7nC6Eo5mQpWnUQVzpe0d9XGS_EFtbZQxFSnDBjtmx8=213)*). If published, this will include your full peer review and any attached files.

If you choose “no”, your identity will remain anonymous but your review may still be made public.*

***Do you want your identity to be public for this peer review?*** *For information about this choice, including consent withdrawal, please see our* [*Privacy Policy*](https://track.editorialmanager.com/CL0/https:%2F%2Fwww.plos.org%2Fprivacy-policy/1/010f019716293912-3089958a-ff36-4e9f-b883-3a4a69a06bd7-000000/YJHYtPCLLVZHaIjK8h2qk8XFnqdrYnkbz_yzkEzMIoQ=213)*.*

*Reviewer #1:* ***Yes:*** *Mohamed Ali Daw, MD, MPS, PhD, MRCPath (1), FTCDI*

*Reviewer #2: No*

*Reviewer #3: No*

*Reviewer #4:* ***Yes:*** *Dr. Fekri Dureab*

*[NOTE: If reviewer comments were submitted as an attachment file, they will be attached to this email and accessible via the submission site. Please log into your account, locate the manuscript record, and check for the action link "View Attachments". If this link does not appear, there are no attachment files.]*

***Figure resubmission:***

*While revising your submission, please upload your figure files to the Preflight Analysis and Conversion Engine (PACE) digital diagnostic tool,* [*https://pacev2.apexcovantage.com/*](https://pacev2.apexcovantage.com/)*. PACE helps ensure that figures meet PLOS requirements. To use PACE, you must first register as a user. Registration is free. Then, login and navigate to the UPLOAD tab, where you will find detailed instructions on how to use the tool. If you encounter any issues or have any questions when using PACE, please email PLOS at* [*figures@plos.org*](mailto:figures@plos.org)*. Please note that Supporting Information files do not need this step. If there are other versions of figure files still present in your submission file inventory at resubmission, please replace them with the PACE-processed versions.*

Answer: These instructions have been noted and addressed as indicated. The figures are compliant with the PACE.

***Reproducibility:***

*To enhance the reproducibility of your results, we recommend that authors of applicable studies deposit laboratory protocols in protocols.io, where a protocol can be assigned its own identifier (DOI) such that it can be cited independently in the future. Additionally, PLOS ONE offers an option to publish peer-reviewed clinical study protocols. Read more information on sharing protocols at* [*https://plos.org/protocols?utm_medium=editorial-email&utm_source=authorletters&utm_campaign=protocols*](https://plos.org/protocols?utm_medium=editorial-email&utm_source=authorletters&utm_campaign=protocols)

Answer: Not applicable.

**References**

1.	Dureab F, Ahmed K, Beiersmann C, Standley CJ, Alwaleedi A, Jahn A. Assessment of electronic disease early warning system for improved disease surveillance and outbreak response in Yemen. BMC Public Health. 2020;20(1):1422. Epub 2020/09/20. doi: 10.1186/s12889-020-09460-4. PubMed PMID: 32948155; PubMed Central PMCID: PMCPMC7501711.
